# Spontaneous clearance of hepatitis C virus after liver transplantation: a report of four cases

**DOI:** 10.1186/s40792-015-0127-0

**Published:** 2015-12-24

**Authors:** Ichiro Tamaki, Toshimi Kaido, Shintaro Yagi, Yoshihide Ueda, Etsuro Hatano, Hideaki Okajima, Shinji Uemoto

**Affiliations:** Division of Hepato-Biliary-Pancreatic Surgery and Transplantation, Department of Surgery, Graduate School of Medicine, Kyoto University, 54 Kawara-cho, Shogoin, Sakyo-ku, Kyoto City, Kyoto 606-8507 Japan; Department of Gastroenterology and Hepatology, Kyoto University, 54 Kawara-cho, Shogoin, Sakyo-ku, Kyoto City, Kyoto 606-8507 Japan

**Keywords:** Hepatitis C virus, Spontaneous clearance, Liver transplantation

## Abstract

End-stage liver disease associated with hepatitis C virus (HCV) infection is the leading indication for liver transplantation. Hepatitis C virus infection recurrence in the graft is common under immunosuppression, leading to an accelerated rate of graft failure. We report the clinical features of four of our patients: three patients presenting with spontaneous hepatitis C virus clearance after liver transplantation and one presenting with transient disappearance of hepatitis C virus postoperatively. The transitional period from surgery to hepatitis C virus clearance was <5 months for all patients. The immunosuppression therapy included tacrolimus, mycophenolate mofetil, and corticosteroids. One ABO-incompatible patient presented spontaneous viral clearance postoperatively for the last 5 years. Two patients had episodes of severe bacterial infection, which resulted in a temporary reduction of immunosuppression. Two patients presented with a transient elevation of transaminase preceding spontaneous hepatitis C virus clearance. These clinical findings suggested that factors including surgical stress, severe bacterial infection, and temporary interruption of immunosuppression were correlated with the reactivation of nonspecific immune responses in the hosts, resulting in spontaneous hepatitis C virus clearance postoperatively.

## Background

Hepatitis C virus (HCV) infection-associated end-stage liver disease is the leading indication for liver transplantation (LT). HCV recurrence in the liver graft is common when HCV viremia is confirmed during LT, leading to an accelerated rate of graft failure [1, 2]. In the liver transplant setting, immunosuppression is an important determinant for increased HCV replication. Therefore, a balance between immunosuppression and anti-viral therapies is crucial in the postoperative clinical course [3].

Spontaneous HCV clearance in chronic carriers of the virus was reportedly consistent in a small post-LT patient population, although the natural course of HCV has not been completely elucidated [4, 5]. Furthermore, spontaneous HCV clearance after LT is rare and few cases have been reported [2]. Here, we report the clinical features of four patients, of whom three patients presented a spontaneous HCV clearance over a short duration after LT (case 1, case 2, and case 3) and one presented transient viral disappearance postoperatively (case 4). These patients were undergoing immunosuppression during HCV clearance.

## Case presentation

### Case 1

**Fig. 1:**
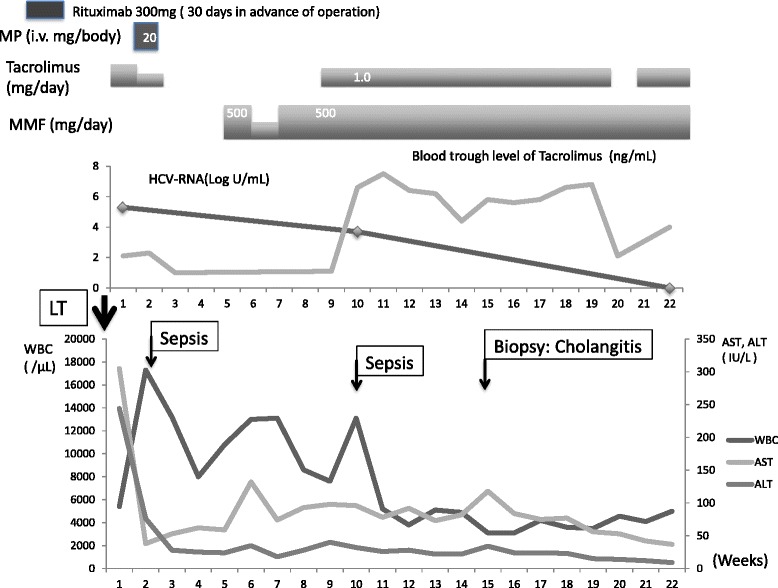
The clinical course of case 1 who is a 66-year-old male. At admission, his HCV-RNA level was 5.3 log IU/ml. HCV-RNA level decreased to 3.7 log IU/ml on day 65 and disappeared by day 152 and remained undetectable over the last 5 years

A 66-year-old man was admitted to our hospital for a living donor liver transplantation (LDLT) for HCV-associated hepatocellular carcinoma (HCC) concomitant with end-stage liver cirrhosis [Child-Pugh score, 9; Model for End-Stage Liver Disease (MELD) score, 21]. Pegylated interferon (PEG-IFN) therapy could not obtain sustained virologic response 8 years before LT. At admission, his HCV-RNA level was 5.3 log IU/ml (range 1.2–8.0 log IU/ml) and genotype was 1b. The donor was ABO incompatible; therefore, plasma exchange and rituximab administration (300 mg/body weight) were conducted before LT. Postoperative immunosuppression included tacrolimus (FK506), mycophenolate mofetil (MMF), and methylprednisolone (MP). Fulminant sepsis associated with blood stream infection and cholangitis made the postoperative course complicated. *Enterobacter cloacae* and *Enterococcus faecium* were detected from the blood culture postoperatively. On day 30, serum aspartate aminotransferase (AST) level increased to 130 U/l, and a liver biopsy was performed. The specimen revealed cholangitis, although acute cellular rejection (ACR) was not biologically confirmed [rejection activity index (RAI) 2]. A similar event was again detected on day 90. HCV-RNA level decreased to 3.7 log IU/ml on day 65 and disappeared by day 152. His HCV-RNA remained undetectable over the last 5 years. To our knowledge, this is the first case of spontaneous HCV clearance for an ABO-incompatible LDLT (Table 1) (Fig. 1).

**Table 1:** Baseline characteristics of the four study patients

	Case 1	Case 2	Case 3	Case 4
Age (years)	66	61	55	55
Sex	Male	Male	Male	Male
MELD score (points)	21.4	11	33	8
Child-Pugh (score)	B (9)	B (9)	C (13)	B (7)
Preoperative HCV-RNA (log IU/ml)	5.3	2.3	2.1	5.5
HCV treatment status	PEG-IFN → nonresponder (8 years in advance of LT)	IFN → terminated due to psychological side effect (21 years in advance of LT)	IFN → failure (9 years in advance of LT)	IFN → nonresponder (8 years advance of LT)
Genotype	1b	2	1b	1b
Rejection episodes	None (RAI 2)	Yes (RAI 3)	None (RAI 1)	Yes (RAI 2)
ABO blood type	Incompatible	Identical	Identical	Identical
Immunosuppression	Rituximab (preoperatively) MMF. FK506, MP	MMF, FK506, MP, PSL	MMF, FK506, MP, PSL	MMF, FK506, MP, PSL
Time to HCV clearance after LT (days)	152	141	177	14
Re-emergence of HCV-RNA	None	None	None	Yes (day 85)
Perioperative morbidity	Sepsis	Sepsis		ACR, steroid pulse
Follow-up periods (years)	5	5	9	1

*MELD* Model for End-Stage Liver Disease, *LT* liver transplantation, *MMF* mycophenolate mofetil, *FK506* tacrolimus, *MP* methylprednisolone, *PSL* prednisolone, *ACR* acute cellular rejection

### Case 2

**Fig. 2:**
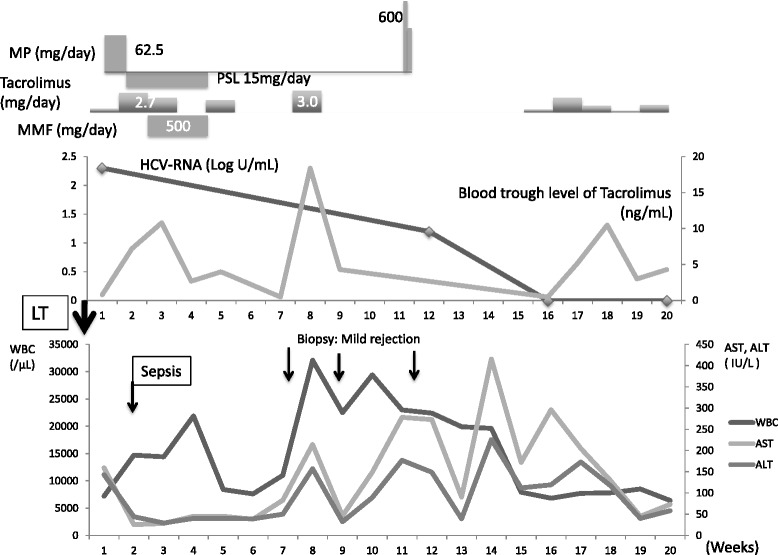
The clinical course of case 2 who is a 61-year-old male. At admission, his HCV-RNA level was 2.3 log IU/ml. On day 112, HCV-RNA level decreased to 1.2 log IU/ml and disappeared by day 141. His HCV-RNA remained undetectable over the last 5 years

Our second patient was a 61-year-old man who underwent LDLT for HCV-associated HCC and end-stage liver cirrhosis (Child-Pugh score, 9; MELD score, 11). His IFN therapy failed 21 years before LT due to a psychological side effect. His HCV-RNA level was 2.3 log IU/ml on admission, and genotype was 2. Severe sepsis occurred on day 13, resulting in a transient reduction of immunosuppression. Gram-negative rods were detected in blood, although the origin was not revealed. On day 39, serum AST and alanine aminotransferase (ALT) levels gradually increased and peaked on day 91 to 416 and 226 U/l, respectively. Liver specimens were taken three times during this period, revealing neutrophil infiltration around the portal area. The diagnosis was mild rejection (RAI 3) and cholangitis. Six hundred milligrams of MP was administered for a treatment of ACR. On day 112, the HCV-RNA level decreased to 1.2 log IU/ml and disappeared by day 141. His HCV-RNA remained undetectable over the last 5 years (Fig. 2).

### Case 3

**Fig. 3:**
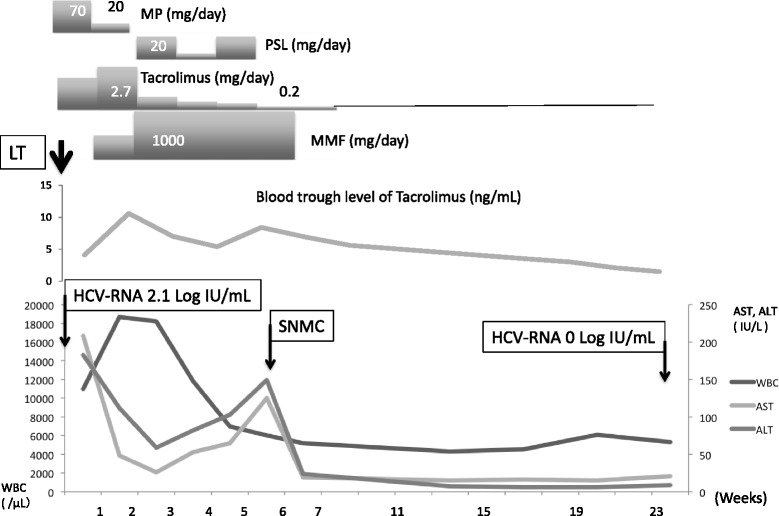
The clinical course of case 3 who is a 55-year-old male. At admission, his HCV-RNA level was 2.1 log IU/ml and disappeared by day 177

Our third case was a 55-year-old man who underwent LDLT for HCC and end-stage liver cirrhosis (Child-Pugh score, 13; MELD score, 33). IFN therapy failed 9 years before LT. His HCV-RNA level was 2.1 log IU/ml, and genotype was 1b. His postoperative course was favorable, and he was discharged from our hospital at 6 weeks after operation. On day 22, his serum AST and ALT levels suddenly increased to 125 and 149 U/l, respectively. A liver biopsy revealed mild lobular inflammation with no evidence of rejection (RAI 1). We commenced strong neo-minophagen C (SNMC) therapy, and the serum transaminase level decreased to the normal range within a few days. During the follow-up period, his HCV-RNA disappeared by day 177 (Fig. 3).

For cases 1, 2, and 3, liver biopsy was performed postoperatively (range 13 months–5 years). All specimens represented no finding of hepatic fibrosis or active inflammation suggesting active viral hepatitis histologically. The transaminase level was within normal range in all of the three patients in the latest blood analysis.

### Case 4

**Fig. 4:**
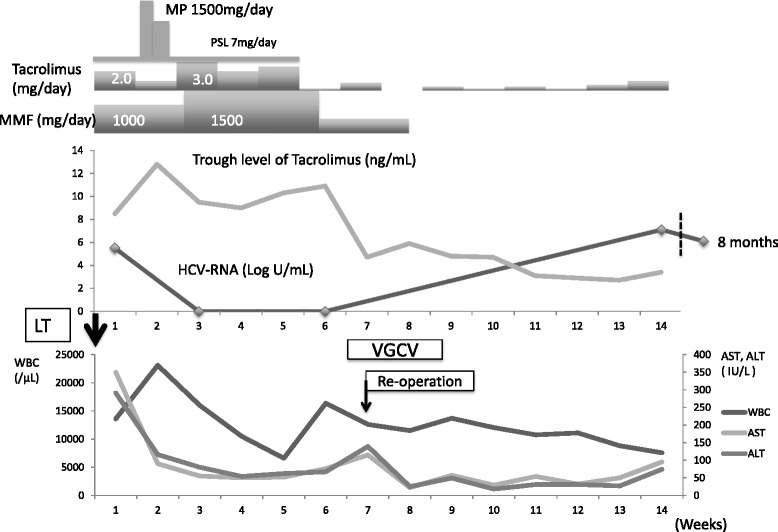
The clinical course of case 4 who is a 55-year-old male. His HCV-RNA level was 5.5 log IU/ml preoperatively. On day 14, his HCV-RNA decreased below the threshold range and remission was confirmed on day 30. The re-emergence of HCV-RNA was confirmed by HCV-RNA levels of 7.1 log IU/ml on day 85 and 6.1 log IU/ml at 8 months after surgery

Our fourth case was a 55-year-old man who underwent LDLT for HCV-associated HCC and liver cirrhosis (Child-Pugh score, 7; MELD score, 8). IFN therapy could not obtain a sustained virologic response 8 years before LT. His HCV-RNA level was 5.5 log IU/ml preoperatively, and genotype was 1b. Preoperative surveillance revealed cytomegalovirus (CMV) antibody (Ab) and human T-cell leukemia virus-1 (HTLV-1) Ab. On day 7, he was diagnosed with ACR because of a sudden elevation in his white blood cell (WBC) count and serum transaminase level. He received a 1500-mg MP bolus for ACR with FK506 and MMF. On day 14 after steroid pulse therapy, his HCV-RNA decreased below the threshold range and remission was confirmed on day 30. During this period, he developed a high fever of undetectable origin and was diagnosed with the reactivation of CMV infection, which was successfully treated with valganciclovir. In addition, two more adverse events occurred postoperatively: one was intra-abdominal bleeding after a liver needle biopsy requiring emergency surgery, and the other was bacteremia because of abdominal abscess formation. After these problems were extricated, the re-emergence of HCV-RNA was confirmed by HCV-RNA levels of 7.1 log IU/ml on day 85 and 6.1 log IU/ml at 8 months after surgery. We have been following up the transition of his HCV-RNA very carefully since then (Fig. 4).

### Discussion

Spontaneous HCV clearance from post-LT patients is an extremely rare phenomenon. For chronic hepatitis C carriers, spontaneous viral clearance is also considered to be a rare phenomenon but exists at a constant rate. Watanabe et al. [4] reported the precise population of spontaneous HCV clearance from a long-term population-based cohort study in which 435 HCV carrier patients were enrolled. He reported that the incidence of spontaneous viral elimination was 0.5 % per person per year (3.7 % individuals during the surveillance period). During January 2005 to December 2014, 156 LT operations were performed for end-stage liver disease or HCV-associated HCC in the Kyoto University Hospital. Three of the patients who presented with postoperative spontaneous viral clearance accounted for 1.9 % of the total.

To investigate the relationship between spontaneous HCV-RNA clearances after LT, we performed a systematic review through PubMed (January 2000 to August 2015) that included keywords such as hepatitis C infection, spontaneous clearance, and LT. We confined the inclusion criteria to shorter interval periods of approximately 1 year after LT. Patients who were on high active anti-retroviral therapy (HAART) for HIV infection or on IFN and ribavirin (RBV) therapies were also excluded. HAART was shown as a factor associated with spontaneous HCV clearance in its natural course [5].

This review revealed a small number of patients (*n* = 8, including our four cases) in four reports, including the present case report [2, 6, 7] (Table 2). The population included six men and two women aged 32–66 years. Each patient had the genotype of type 1 (including type 1b in three patients), two patients had type 2, and one patient had type 4. Postoperative viral loads were 4.3 ± 1.13 log IU/ml [mean ± standard deviation (SD)]. Focusing on the particular perioperative events, six patients presented a transient elevation of the serum transaminase level before HCV clearance (AST, 130–532 U/ml; ALT, 100–540 U/ml; interval periods, 17–280 days). Four patients had episodes of ACR; several patients were treated with immunosuppressive agents including steroids before HCV clearance. Two of our patients suffered severe septic complications during their perioperative periods. HCV clearance was shortly confirmed after the resolution of these concomitant problems. These episodes suggest an association between extraordinary perioperative fluctuations of the host immunity and viral clearance. Further, over a natural course of HCV infection, several articles described spontaneous clearance together with a significant clinical event such as immune reconstitution after HAART, immunosuppressive therapy termination, pregnancy, or other viral infection onset [8, 9].

**Table 2:** Case reports of patients showing spontaneous HCV clearance within 1 year of liver transplant without postoperative anti-viral therapy

Author (published year)	Age (years)	Sex	Preoperative HCV-RNA (log IU/ml)	HCV genotype	HCV treatment status	Rejection episodes	Immunosuppression	Time to HCV clearance after LT (days)	Postoperative morbidity	Transaminase elevation ahead of HCV clearance
Doughty AL [6] (2000)	49	Male	5.7	N/A	N/A	Yes	MP, AZA, CSA	383	N/A	Yes
Elsiesy H [2] (2015)	32	Female	4.8	4	Failure	None	FK506, CSA	30	N/A	Yes
Kogiso T [7] (2015)	50	Female	4.3	1	Naïve	Yes	FK506, MMF, MP, CS	87	ACR	Yes
	52	Male	4.8	2	Naïve	None	FK506, MMF, MP, CS	115	N/A	Yes
Our cases										
Case 1	66	Male	5.3	1b	Failure	None	Rituximab (preoperatively) FK506, MMF, MP, PSL	152	Sepsis	Yes
Case 2	61	Male	2.3	2	Failure	Yes	FK506, MMF, MP, PSL	111	Sepsis, ACR	Yes
Case 3	55	Male	2.1	1b	Failure	None	FK506, MMF, MP, PSL	177	None	No
Case 4	55	Male	5.5	1b	Failure	Yes	FK506, MMF, MP, PSL	14	ACR	No

*LT* liver transplantation, *N/A* not available/data not reported, *AZA* azathioprine, *CSA* cyclosporine, *MMF* mycophenolate mofetil, *FK506* tacrolimus, *MP* methylprednisolone, *PSL* prednisolone, *ACR* acute cellular rejection

In chronic HCV infection, the liver is infiltrated by mononuclear cells including CD4+, CD8+ T lymphocytes, B lymphocytes, natural killer (NK) cells, and NK T cells [10]. Chronic HCV infection is characterized by an impaired HCV-specific cytotoxic T lymphocyte (CTL) response that is unable to control HCV replication [11]. Mutations in the hypervariable region-1 of HCV-envelope glycoproteins and quasispecies enable HCV to escape T-cell responses and immunological neutralization [8, 10]. The mechanism of host immunity restoration against HCV, which might be a critical part of spontaneous HCV clearance, remains unclear. Lauer and Kim [8] propounded two conceivable scenarios for improved anti-HCV immunity: one was CD4+ and CD8+ T-cell reactivation, and the other was a massive release of type 1 IFN, which activates the dormant innate immune response. We speculate that factors including operative physical stress, severe postoperative infection, and temporary immunosuppression interruption were correlated with the reactivation of nonspecific immune responses. Activation of Toll-like receptors, which recognize nonspecific molecular structures on microorganisms and induce type 1 IFN responses, is expected to be a new therapeutic strategy for chronic HCV infection [12]. We speculate that a similar mechanism explains our findings. Hence, the transient elevation of transaminase detected before HCV clearance might reflect the response of CTLs against infected hepatocytes.

One patient who presented transient viral disappearance had HCV re-emergence immediately following reoperation and intra-abdominal abscess formation. CMV reactivation was confirmed at the same time. An intermediate replicative form of the HCV genome can persist for many years at very low levels in peripheral mononuclear cells after an apparently complete resolution of chronic hepatitis C [5]. We speculate that further immunosuppressive conditions induce HCV re-emergence, suggesting that the transient disappearance of HCV occurs in more cases. In our clinical practice, we routinely evaluate the serum HCV-RNA level within a few weeks after LT.

## Conclusions

We reported three cases of spontaneous hepatitis C virus clearance after liver transplantation and one presenting with transient disappearance of hepatitis C virus postoperatively. We believe that an accidental immunity fluctuation in the hosts resulted in spontaneous HCV clearance after LT. Further studies are necessary to ascertain the factors that may facilitate HCV clearance.

## Consent

Informed consent was obtained from the patients. The figures related to the article do not contain any information that may affect the patient’s privacy in any way.

## Abbreviations

Ab: antibody
ACR: acute cellular rejection
ALT: alanine aminotransferase
AST: aspartate aminotransferase
AZA: azathioprine
CMV: cytomegalovirus
CSA: cyclosporine
CTLs: cytotoxic T lymphocytes
FK506: tacrolimus
HAART: high active anti-retroviral therapy
HCC: hepatocellular carcinoma, HCV, hepatitis C virus
HTLV-1: human T-cell leukemia virus-1
IFN: interferon
LDLT: living donor liver transplantation
LT: liver transplantation
MELD: Model for End-Stage Liver Disease
MMF: mycophenolate mofetil
MP: methylprednisolone
N/A: not available/data not reported
PSL: prednisolone
RAI: rejection activity index
RBV: ribavirin
SD: standard deviation
SNMC: strong neo-minophagen C
VGCV: valganciclovir
WBC: white blood cell
